# Prevalence of Workplace Sexual Violence against Healthcare Workers Providing Home Care: A Systematic Review and Meta-Analysis

**DOI:** 10.3390/ijerph17238807

**Published:** 2020-11-27

**Authors:** Marco Clari, Alessio Conti, Alessandro Scacchi, Marco Scattaglia, Valerio Dimonte, Maria Michela Gianino

**Affiliations:** Department of Public Health and Paediatrics, University of Torino, via Santena 5bis, 10126 Turin, Italy; marco.clari@unito.it (M.C.); alessio.conti@unito.it (A.C.); marco.scattaglia@unito.it (M.S.); valerio.dimonte@unito.it (V.D.); mariola.gianino@unito.it (M.M.G.)

**Keywords:** sex offenses, healthcare workers, home care, systematic review, meta-analysis

## Abstract

This systematic review and meta-analysis sought to explore the prevalence of sexual violence including both sexual harassment and abuse, perpetrated by clients against home healthcare workers (HCWs), including professional and paraprofessional HCWs. To this end, we systematically searched five relevant databases. Two reviewers extracted data from the included studies independently and performed a quality appraisal. Overall and subgroup random-effects pooled prevalence meta-analyses were performed. Due to high heterogeneity, a more robust model using a quality effect estimator was used. Fourteen studies were included, and the prevalence of sexual violence was 0.06 (95% confidence interval (CI): 0.01–0.13). Paraprofessionals had a higher prevalence of sexual violence (0.07, 95% CI: 0.00–0.18 vs. 0.05, 95% CI: 0.00–0.12), and the prevalence of sexual abuse was lower than that of sexual harassment (0.04, 95% CI: 0.00–0.10 vs. 0.10, 95% CI: 0.03–0.18). This systematic review estimated the prevalence of sexual violence across home HCWs from different high-income countries, highlighting the presence of this phenomenon to a lesser but nevertheless considerable extent compared to other healthcare settings. Health management should consider interventions to prevent and reduce the risk of home HCWs from being subjected to sexual violence, as the home-care sector presents particular risks for HCWs because clients’ homes expose them to a relatively uncontrolled work environment.

## 1. Introduction

Workplace violence is a severe occupational issue that has been studied from different points of view, such as the many manifestations of workplace violence, how it varies across different occupations, and whether and how the victims’ experiences differed by perpetrator characteristics [1]. The prevalence of workplace violence is highly dependent upon the work sector and job characteristics. In particular, healthcare workers (HCWs) are more likely to experience workplace violence and injuries during their job than other industry workers [2,3,4].

Previous reviews were focused on violence perpetrated towards specific groups of HCWs (i.e., physicians, nurses, dental hygienists, or emergency medical service personnel) [5,6,7]. Moreover, most of the studies conducted to date have only addressed workplace violence in the hospital setting [8,9,10]; therefore, the reviews previously undertaken have considered single wards or divisions, such as emergency departments, psychiatric hospitals, and geriatric wards, where workplace violence exposure is of particular concern [7,11]. Only a few researchers have examined workplace violence against HCWs in non-hospital settings [12,13,14], especially against HCWs providing home care.

Home care is a way to provide care and social assistance directly at the patients’ home, rather than in the hospital or a long-term care facility. It can be provided by professional and paraprofessional HCWs, and it consists of a range of medical, nursing, and hands-on care, such as physical care (e.g., toileting, mobilization, and feeding), the provision of nursing techniques (e.g., medication administration, blood sampling, and device positioning and management), medical examinations, and palliative care [15]. Home-care patients can be people with a wide range of needs, such as short-term care for acute conditions, long-term care for chronic conditions and disabilities, and other specific needs, such as end-of-life care or rehabilitation [16]. Home care has been associated with decreased mortality [17,18], improved quality of life [12], and reduced hospitalization rates [13]. Moreover, rather than hospitalization and long-term care, home care may represent a cost-effective alternative for people living in rural areas and for those living without family support or a caregiver [19].

The home-care sector presents particular risks for HCWs because clients’ homes expose HCWs to a relatively uncontrolled work environment [20]. The isolated nature of HCWs performing their job at clients’ homes could represent a further risk of violence from both patients and their relatives [21,22]. Moreover, the protections that HCWs usually have in the hospital setting, such as the presence of co-workers or security guards, are not present during home care. HCWs providing home care cannot control their work environment; consequently, in the absence of employer safety policies and programmes, home HCWs must rely on their own resources to deal with abuse and violence [23].

Workplace violence has a relevant impact on HCWs’ wellbeing; in particular, it contributes to negative psychological outcomes, such as depression [24], shame, anxiety and anguish, anger, and post-traumatic stress disorder [25]. Furthermore, workplace violence affects not only the individual HCW’s job processes, but also the whole work organization. Indeed, the negative effects of workplace violence influence the organization’s well-being, in particular increasing occupational stress and burnout, job disruption, absenteeism, and HCWs’ intention to leave their job or position [26,27].

Workplace violence is a broad definition that includes a spectrum of physical, verbal, emotional, and sexual behaviours [28]. In particular, sexual violence can range from verbal sexual interaction to physical assaults. Verbal sexual interactions are often defined as sexual harassment and include behaviours, such as flirting and teasing [29], but workers’ gender, age, ethnicity, and place of living contextualize the definition of what is considered sexual violence or harassment [30]. Estimating the extent of this phenomenon in the home-care setting is currently extremely challenging. Indeed, to date, no systematic reviews have investigated the prevalence of sexual violence in this context. Despite the relevance of this phenomenon, sexual violence perpetrated by clients in a home-care setting is an issue that has received considerably less attention than physical and non-physical violence against HCWs across other clinical settings, limiting the possibility of estimating the magnitude of this phenomenon.

Thus, the aim of this review was to explore the prevalence of sexual violence, including both sexual harassment and abuse, perpetrated by clients against home HCWs, including professional and paraprofessional HCWs.

## 2. Materials and Methods

This systematic review and meta-analysis was reported following the Preferred Reporting Items for Systematic Reviews and Meta-Analyses (PRISMA) guidelines. The protocol of this systematic review was preregistered on Prospero (https://www.crd.york.ac.uk/PROSPERO; registration number: CRD42020162855). This article presents aggregate data from primary studies maintaining the same objective as the original studies; thus, no ethical approval was requested.

### 2.1. Inclusion and Exclusion Criteria

To be included in this systematic review, studies were required to be primary investigations based on a sample of HCWs involved in the home care of people requiring healthcare assistance. In this systematic review, ‘healthcare workers’ is a comprehensive term including professional HCWs, such as physicians, registered nurses, allied health professionals (e.g., physiotherapists, social workers, and dietitians), and paraprofessional workers. Paraprofessional refers to paid employees providing direct care to elderly and disabled people, such as home health aides, personal care workers, and nurse-assistants or nurse aides. We did not restrict our search for HCW age, gender, ethnicity, or country in which the primary research was conducted.

All original articles reporting the exposure to sexual violence by a patient or any other person in the home where HCWs performed their job were included (i.e., type II violence). We considered both sexual harassment and abuse. Sexual harassment includes any kind of unwanted sexual proposition or attention, including but not limited to humiliating or offensive expressions, comments with sexual overtones, lascivious or unpleasant looks, indecent acts, and requests to touch or grab the other person’s body [31]. Sexual abuse includes any indecent physical assault, ranging from brushing, touching, or grabbing genitalia to forced sexual acts and rape [31].

Only sexual violence occurring in the home-care setting was considered in the present systematic review. The main outcomes considered were the sexual violence prevalence rate and sexual violence characteristics.

All professional and paraprofessional HCWs, as previously defined, employed in the hospital setting (i.e., general and specialty) were excluded. Type I and III violence were not considered. Primary care, such as nursing homes or mental healthcare centres, as well as out-of-hospital emergency settings, were also excluded. Articles related to any other type of violence and published in non-peer reviewed journals were excluded. We set no limit on publication date.

Observational studies were included in this systematic review, including, cohort studies, case-control studies, and cross-sectional studies. Moreover, descriptive observational study designs, including case series, individual case reports, and descriptive cross-sectional studies, were also considered. Qualitative studies were excluded. The quantitative results of mixed methods studies were included if it was possible to extract data. 

### 2.2. Search Strategy

An initial search was performed on PubMed to address the most appropriate terms to adopt in the search. Then, PubMed, Cumulative Index to Nursing and Allied Health Literature (CINAHL), APA Psycinfo, ISI Web of Science, and Embase Database were systematically searched from inception to February 2020. The main search terms used in the various databases were aggression, violence, workplace violence, abuse, occupational injuries, healthcare workers. To explore the home-care context the following specific terms were used: home care, home therapy, home community care, home-care services, and home nursing. Thesaurus and free terms were combined to include all the possible terms related to the phenomenon. As an example, the search conducted on PubMed is reported as Supplementary Material (Table S1). Included studies were identified by searching the electronic databases and scanning the reference lists of relevant articles. An expert librarian was involved in the search.

First, the records obtained were checked for duplicates, and then the titles and abstracts of the identified citations were screened against the inclusion and exclusion criteria by two independent reviewers. The full texts of potentially relevant papers were read in full and screened for final inclusion by two independent reviewers. Reasons for exclusion were recorded transparently. Any disagreements that arose at any stage of the review were resolved by discussion with a third reviewer.

### 2.3. Data Extraction and Quality Assessment

Data were extracted by two reviewers independently. Data were checked by a third reviewer, and 100% agreement was reached. The data extracted included the author(s), year of publication, title of the paper, country in which the study took place, details about the condition, study design, study sample, and prevalence rates of sexual violence.

A quality score was calculated for the included studies. This quality score assessed each paper against six predefined lists of bias. These biases included (1) well-defined observation period (i.e., yes, no), (2) use of standardized diagnostic criteria (i.e., diagnostic system report, own system or not specified), (3) manner in which the cases were ascertained (i.e., community survey, case registers, and not specified), (4) way in which the data were obtained (i.e., administered interview, systematic casenote review, case records, and not specified), (5) extent to which the sample was representative of the area of interest (i.e., broadly, small area, and convenience sampling), and (6) the prevalence measure used (i.e., point, 12-month, and lifetime prevalence). This quality score is essentially a safeguards score. The score could range from 0 to 11, with higher scores representing studies with higher quality. The scores were then converted, with the best study ranked 1 and the other studies with lower scores ranked lower. This rescaled quality rank has a monotonic relationship with the bias interclass correlation and helps reduce the estimator variance [32,33]. Quality was assessed by two authors independently, and any disagreement was solved by discussion.

### 2.4. Statistical Analysis

#### 2.4.1. Overall Pooled Prevalence of Sexual Violence against Home-Care Healthcare Workers (HCWs)

Before performing the overall pooled prevalence meta-analysis, the heterogeneity of prevalence estimates were assessed by calculating the I2 index and performing the Cochran Q test. An I^2^ > 50% and Cochran Q test *p*-values < 0.05 represented a high degree of significant heterogeneity. Due to the high heterogeneity found and expected, we performed a random-effects meta-analysis of sexual violence with 95% confidence intervals (CIs). As in highly heterogeneous meta-analyses, the random-effects model does still have a high mean squared error, a meta-analysis using a quality effect estimator was also performed. This model is more robust and leads to maintenance of the correct coverage probability of the confidence interval, regardless of the level of heterogeneity. Quality assessed through the six items of the quality score is normalized ranging from 0 to 1. Additionally, as the quality index increases, the confidence interval width around the pooled estimate decreases. To synthetize proportions retrieved from the studies included, due to the non-normally distributed data, the Freeman-Tukey double arcsine transformation was used. This method was chosen to stabilize the data variance [34].

#### 2.4.2. Subgroup Meta-Analysis of the Prevalence of Sexual Violence among Home-Care HCWs

We conducted subgroup meta-analyses to determine potential sources of heterogeneity. Three subgroup meta-analyses were performed: one assessing the prevalence of sexual violence during the last 12 months and lifetime, one assessing the prevalence of sexual violence between professional and paraprofessional HCWs, and the other assessing the prevalence of the two types of sexual violence in the study (i.e., sexual harassment and sexual abuse). Data from at least three studies should be available to perform subgroup analyses. 

To evaluate the association of HCW characteristics with sexual violence, a meta-regression was also performed. Data on HCW characteristics from at least 10 studies should be available to perform a univariate meta-regression and 20 for a multivariable meta-regression.

Analyses were conducted using the MetaXL version 5.3 (Epigear International Pty Ltd., Sunrise Beach, Australia) [33].

## 3. Results

**Figure 1:**
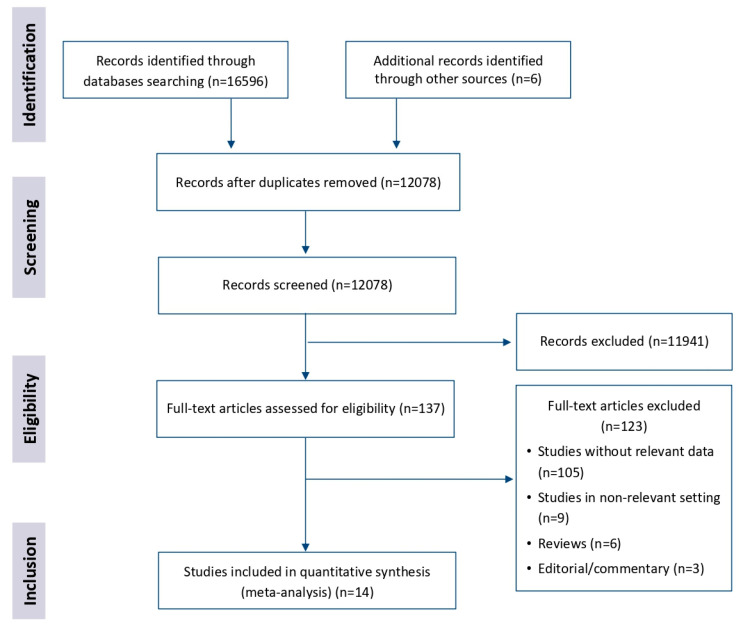
Literature search flow.

A total of 14 studies were included in the systematic review, and the screening process is presented in Figure 1.

The studies were published from 2001 to 2019, and all used a cross-sectional design. The total sample included in the meta-analysis was 6014 (range 35–1249), and most of the HCWs included were women (90.7%) with a mean age of 39.4 (±9.6) years. The mean job seniority was 10.5 (±7.5) years. Three studies included only females. Seven studies reported sexual violence for professional HCWs, with the nursing profession being the most heavily investigated. Paraprofessional HCWs were included in six studies. All studies were conducted in high-income countries, specifically five in the US, three in Israel, two in Canada and Japan, and one each in Australia and Sweden. The characteristics of the included studies are reported in Table 1.

The overall quality of the studies was high (Table 1). The complete quality assessment is reported in Supplementary Material (Table S2).

As all the meta-analyses had overall very high heterogeneity (I^2^ ≥ 95%; Cochran Q, *p* ≤ 0.001), meta-analyses by study quality effect were reported.

**Figure 2:**
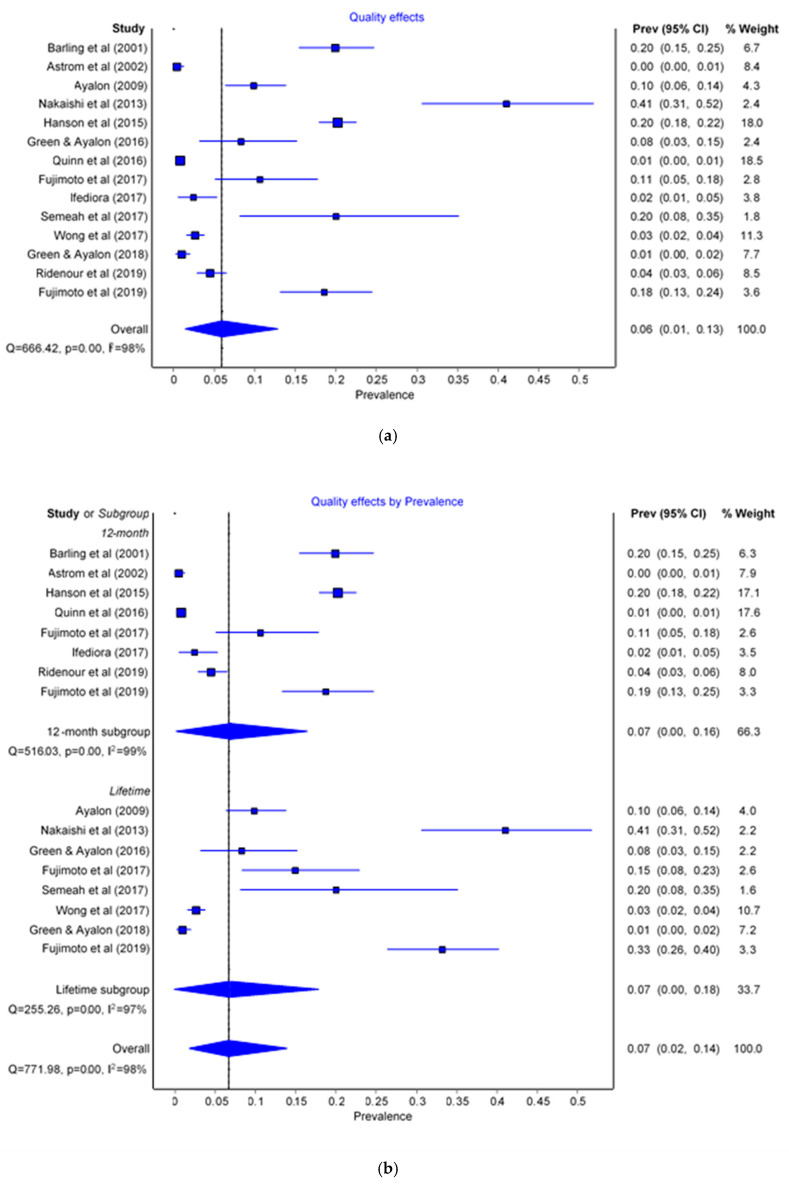

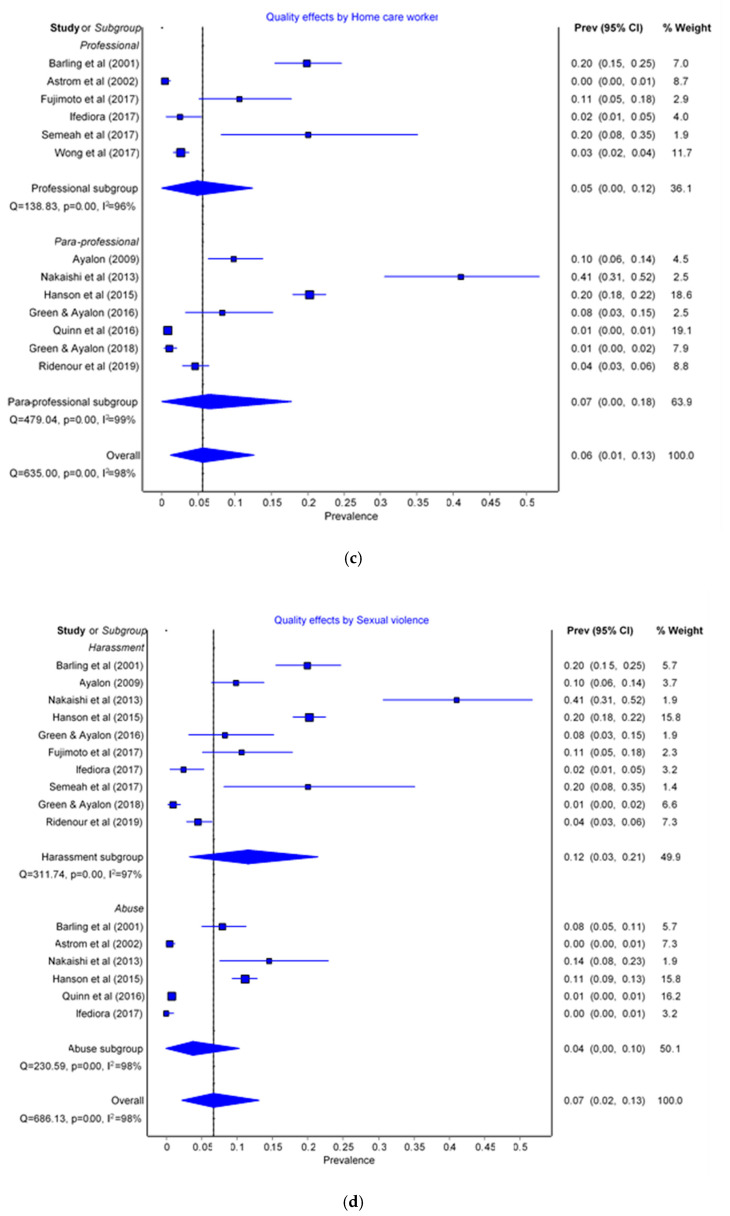
Forest plots of the prevalence of sexual violence for (**a**) overall, (**b**) type of prevalence (12 months vs. lifetime), (**c**) type of worker (professional vs. paraprofessional), and (**d**) type of sexual violence (harassment vs. abuse).

The prevalence of sexual violence (both harassment and abuse) among HCWs (both professional and paraprofessional) was 6% (95% CI: 0.01–0.13) with an I^2^ = 98% (Figure 2a).

Eight studies evaluated 12-month prevalence, and eight lifetime prevalence. There was no difference for 12-month (prevalence = 7%, 95% CI: 0.00–0.16) or lifetime prevalence (prevalence = 7%, 95% CI: 0.00–0.18) for sexual violence (Figure 2b). The heterogeneity of the 12-month prevalence was 99%, while in the lifetime subgroup it was 97%.

Professional HCWs were studied in six studies while paraprofessionals were studied in seven. Paraprofessional HCWs had a higher prevalence of sexual violence (prevalence = 7%, 95% CI: 0.00–0.18) than professional HCWs (prevalence = 5%, 95% CI: 0.00–0.12) (Figure 2c). For the professional subgroup the meta-analysis heterogeneity was 96% and 99% for the paraprofessional subgroup.

Most studies investigated sexual harassment (n = 10), while sexual abuse was less investigated (n = 6). The prevalence of sexual abuse was lower (prevalence = 4%, 95% CI: 0.00–0.10) than that of sexual harassment (prevalence = 12%, 95% CI: 0.03–0.21) (Figure 2d). I^2^ values were 97% and 98% for the harassment and abuse subgroups meta-analyses respectively.

Due to the lack of data reporting personal and working characteristics related to workplace sexual violence, no meta-regression could be performed.

The event rates of the included studies are scattered (Figure 2a). Moreover, the funnel plot and the Doi plot detected major asymmetry, representing the possible presence of reporting bias. The plots are shown as a Supplementary Material (Figure S1). Sensitivity analysis using random effects models detected no impact of the method used. Also, sensitivity analysis excluding studies with bigger sample size showed no impact on the estimates, thus resulting in robust results.

## 4. Discussion

The results of this systematic review and meta-analysis estimated the prevalence of sexual violence across home HCWs from different high-income countries, highlighting the presence of this phenomenon to a lesser but nevertheless considerable extent compared to other healthcare settings. There was no difference in the 12-month and lifetime prevalence of sexual violence, although paraprofessional HCWs reported a higher prevalence of this phenomenon than did professional HCWs. Furthermore, the prevalence of sexual abuse was lower than that of sexual harassment, which affected one out of 10 home HCWs.

Our results contribute to filling a literature gap, enlightening the home-care perspective, focusing on a different setting than hospitals and other primary care facilities where sexual violence prevalence is considerably higher. In this regard, according to Spector et al., a significant difference in the sexual violence rate was found in different contexts, with sexual harassment rates varying from 1.2% in geriatric units to 29.7% in psychiatric wards and 41.1% in general samples [7]. Although the results obtained would seem to minimize sexual violence against HCWs in home care, there are some contextual factors specific to this setting that need to be considered when interpreting the results obtained.

Women made up the majority of our sample (90.7%), and nurses were the most investigated HCW profession. This result might be justified due to the high prevalence of women in the health and social workforce, accounting for over 70% globally, particularly in the nursing profession [35]. That gender imbalance is of utmost relevance when sexual violence is studied, as in settings where women are more represented, nurses are particularly at risk for sexual harassment in the workplace [36]. In particular, compared to their male colleagues, female nurses are more likely to be sexually harassed at work by patients, their families, or colleagues [37]. Moreover, gender-based violence represents a global public health issue that arises across geographical and cultural contexts and constitutes a broad societal problem [38].

The first identified source of sexual harassment against female nurses is clients and their family members [36], whereas the second source of such violence is represented by physicians followed by other HCWs [39,40]. Hence, the majority of potential perpetrators of violence towards nurses is their colleagues. As only clients and their family members represent a potential source of sexual violence in home care, it is expected that the prevalence of sexual harassment identified would be lower than those reported in other contexts. In this regard, the lack of other HCWs during home-care practices, representing potential sexual violence sources, might constitute a reason for the lower prevalence of sexual harassment found in this review.

The prevalence of sexual harassment among home HCWs is lower than that observed in other contexts. The estimated worldwide prevalence of sexual harassment among nurses has indeed been estimated to be 12.6% over the last 12 months and 53.4% during their careers [37]. Verbal abuse, including sexual harassment and innuendo, was the most common form of aggression reported by the majority of these HCWs in any environment [41]. In particular, female nurses were most commonly sexually harassed, with a percentage of 43.15%, as found in other studies with a prevalence higher than half the sample [40,42,43,44,45].

Findings from our review showed that among home HCWs, the prevalence of sexual abuse is lower than that of sexual harassment. Although the prevalence of sexual abuse has been determined to be limited, it is clearly not negligible, suggesting the need for increased safety measures towards home HCWs. This setting, mostly represented by the patients’ house, is not neutral, as it only includes the patients, their relatives, or their caregivers. Furthermore, the home setting does not provide surveillance guards, cameras, and other people to seek help in the case of danger, suggesting to the perpetrator of violence that they are safe and should feel confident in performing acts of violence.

An additional consideration in evaluating the obtained findings is related to the underreporting of workplace violence. This phenomenon is common among HCWs (46% to 80%) [46]. Several reasons for not reporting sexual violence have been found in the literature, such as fear of being punished or ridiculed and lack of trust in investigators, police, and other health workers [17] as well as shame and embarrassment [47]. Additionally, the lack of consequences following a sexual harassment report could act as a deterrent to denouncing it [48]. Home care is led by HCWs acting alone, thereby hindering the possibility of reporting violence experienced because of the lack of witnesses within their work area, which is the patient’s home. Moreover, nurses’ tendency to hesitate to stop or report inappropriate behaviours by patients leads to a reduction in requests for help to colleagues or supervisors and measures against violence [49]. Therefore, the real prevalence of sexual violence may be considerably higher than that observed in this meta-analysis.

Patients receiving home-care services are often affected by terminal illnesses, disabilities, or dementia [50]. Given that these patients’ physical and mental states are severely compromised, they could more frequently engage in verbal aggression or aggressive behaviours rather than sexual abuse [51]. Furthermore, considering the severe burden of disease experienced by the most severely ill patients, HCWs could accept their violent conduct as part of their job, judging it as normal, resulting in fewer aggression reports and acceptance of these struggles. This tendency to accept violence has already been reported in other studies describing the underreporting or neglect of sexual violence by HCWs, especially among females [52,53].

This study reported no differences in the prevalence of sexual violence between the 12-month observation and lifetime periods. The absence of a difference in the prevalence of sexual violence when examining the entire HCW career could suggest that such a phenomenon is rare during home care. However, this finding contrasts with a study that found a career effect resulting in a higher prevalence of sexual violence experienced by HCWs during their entire working experience [7]. In this regard, the reported rates of sexual violence are considerably higher when the examined timeframe is the whole working career, rather than a more limited timeframe, such as the last 12 months. Furthermore, the low prevalence of sexual violence found in this study may also be attributable to the tendency to assign significance to persistent non-consensual sexual harassment [54]. Thus, occasional sexual harassment could not be fully reported by participants in the included studies, limiting this phenomenon’s real impact on the HCW population.

The findings described in this review showed a higher prevalence of sexual violence reported by paraprofessional HCWs in home care. Sexual violence has been described as a transversal phenomenon, primarily affecting nurses but also affecting doctors, nurse aides, and all those involved with the clients. In this regard, a study showed that half of female medical students and a third of female doctors reported sexual harassment during their work. In a home-care setting, paraprofessional HCWs spend the majority of time with patients. Therefore, they can be expected to be more affected by sexual violence than other HCWs. Moreover, the lower authority of paraprofessional HCWs perceived by clients could act as a factor influencing the prevalence of violence experienced in this group [41]. Paraprofessional HCWs are more involved in direct care activities than clinical consultations, which are usually performed by doctors. In this regard, a review highlighted that the most common time for aggression is represented by hands-on care to patients. Furthermore, miscommunication was identified as a potential trigger for aggressive incidents from patients [41]. Standard nursing practices, such as personal care, hygiene, and wound dressing, have been identified as potentially at risk of triggering aggressions [55,56]; thus, they could represent occasions of sexual violence. In this regard, Boyd found that nurse aide compensation claims for workplace violence accounted for the second-highest expense for HCWs [57].

The findings may serve as a guide for health policymakers in their efforts to prevent the occurrence of sexual violence among home-care HCWs. The consequences of this phenomenon might have a major impact on healthcare services. Sexual harassment leads to severe mental (e.g., anxiety, depression, and stress) and physical (e.g., headache, exhaustion, gastritis, nausea or vomiting, weight gain or weight loss, muscle pain or convulsion, and dizziness) effects, as well as sleep disturbances [36]. Following an aggressive incident, if nurses are considered, they often report experiencing sadness, shock, confusion, anger, and embarrassment [41]. In particular, sexual harassment towards HCWs leads to high stress, poor mental health and quality of life, burnout, job dissatisfaction, and poor work quality and efficiency [37]. Although sexual abuses have been identified as less prevalent than sexual harassment in home HCWs, their consequences have a severe impact on the mental and physical health of those who suffered them. These include injuries, sexually transmitted infections, and unwanted pregnancy [58]. Furthermore, sexual violence has been found to represent a consistent cause of post-traumatic stress disorder in women and leads to a wide range of possible psychological consequences, such as anxiety, depression, and suicidal ideation [59]. If not addressed, these consequences might constitute significant welfare problems due to HCWs’ job withdrawals and potential social losses, as observed in the general population. In addition to the direct impact on HCWs, their families and clients’ repercussions must be considered [60].

Health management should consider interventions to prevent and reduce the risk of home HCWs from engaging in sexual violence. Early warning systems should be implemented to educate home HCWs in recognizing potential at-risk situations and responding effectively to limit the possibilities of assaults. Despite the significant variability in the prevalence reported in the included studies, this phenomenon represents a severe problem, even in home care, which should be reported promptly, but may often be underreported. Therefore, more accessible reporting methods combined with a cultural approach encouraging the detection of sexual violence suffered by home HCWs are needed to improve the estimation of the prevalence and give it a correct dimension. International guidelines indicate that victims of sexual violence should receive forensic, clinical, and psychological care both in acute and follow-up care. Hence, a regular clinical and psychological follow-up programme should be planned for home HCWs who have experienced sexual violence. Those measures would be effective in supporting them in overcoming those traumatic events and avoiding their constituting a source of deprivation for themselves, their families, and the healthcare system.

### Strengths and Limitations

This review presents a number of limitations. First, the studies included were conducted in high-income countries, limiting the generalizability of the findings to other medium- and low-income countries. Second, the review of five databases and the restriction to studies published in English and Italian could have excluded relevant papers. Nevertheless, the application of a systematic approach and inclusion of two independent reviewers and an expert librarian in the search, screening, and extraction phases, contributed to reducing biases.

Another limitation of this review was the inconsistency across the included studies in operational definitions and way of measuring sexual violence. Sexual violence included non-physical violence, such as sexual innuendo and flirting, as well as the physical form of sexual violence. Moreover, to capture physical sexual violence differently, the terms “sexual aggression,” “sexual abuse,” and “sexual assault” were used. If the distinction between sexual harassment and abuse was not clear from original studies, violence was categorized on a discretional basis. Researchers generally agree that there is not a single, unifying theoretical framework or definition for sexual violence [51]. Thus, future consensus on the definition of sexual violence could contribute to increasing the homogeneity among measurements in different countries and contexts.

Some studies included used self-reported measures. This could contribute to an underestimation of the phenomenon due to resistance to reporting sexual violence because of shame and fear of being doubted. Furthermore, the use of lifetime prevalence in the same studies could have led to recall bias.

This inconsistency in the definitions, measurements, populations, and type of prevalence measured contributed to the high meta-analysis heterogeneity. To limit this problem, we used a double arcsine transformation aimed at stabilizing the prevalence variance [61]. Moreover, all the meta-analyses performed used a quality effect estimator. This method is preferable to random-effects models because, unrelated to the level of heterogeneity, it retains a lower observed variance while maintaining the correct probability of the confidence interval. Moreover, quality effects are more robust to subjectivity due to completely random entry [62].

Despite the aforementioned limitations, this systematic review is the first to specifically focus on sexual violence in home care using robust meta-analysis methods.

## 5. Conclusions

This systematic review and meta-analysis showed that the prevalence of sexual violence against home HCWs is lower than that in other clinical settings for both 12-month and lifetime prevalence. As expected and hoped, the prevalence of sexual abuse was lower than that of sexual harassment. Finally, paraprofessional workers had a higher prevalence than professional workers. Our results indicate that even with a lower prevalence, sexual violence against home HCWs is an issue that must be assessed and contrasted with the high psychological impact on workers. Further studies using unambiguous definitions and measurements should be conducted. Systematic monitoring should be conducted to prevent and reduce the risk of home HCWs from engaging in sexual violence, and specific psychological support for assaulted HCWs should also be implemented.

## Figures and Tables

**Table 1:** Included studies’ characteristics.

Author(s), Year	Country	Prevalence Measure	Population	Sample	Gender	Mean Age [years] (SD)	Mean or Median Seniority [Years] (SD - Range)	Quality Assessment [0–11]
Barling et al., 2001	Canada	Point prevalence (6-month)	*Professionals* (Nurses, social workers)	292	F = 292; M = 0	36.6	8	11
Astrom et al., 2002	Sweden	12-month prevalence	*Professionals* (Nurses)	506	F = 463; M = 43	40.5 (±9.3)	NA ^§^	9
Ayalon, 2009	Israel	Lifetime prevalence	*Paraprofessionals* (Home healthcare aides)	245	F = 204; M = 41	36.2 (8.1)	NA	8
Nakaishi et al., 2013	US	Lifetime prevalence	*Paraprofessionals* (Home healthcare aides)	83	F = 83; M = 0	NA	9.8 (9.2)	8
Hanson et al., 2015	US	12-month prevalence	*Paraprofessionals* (Home healthcare aides)	1214	F = 1214; M = 0	47.3 (13.8)	7.9 (7.3)	9
Green and Ayalon., 2016	Israel	Lifetime prevalence	*Paraprofessionals* (Home healthcare aides)	85	F = 73; M = 12	37.0 (6.7)	NA ^§^	8
Quinn et al., 2016	US	12-month prevalence	*Paraprofessionals* (Home healthcare aides)	1249	F = 1086; M = 159 *	NA ^§^	NA	9
Fujimoto et al., 2017	Japan	12-month + lifetime prevalence	*Professionals* (Nurses)	94	F = 73; M = 21	46.1 (9.1)	19.8 (3.7–42.6)	9
Ifediora, 2017	Australia	12-month prevalence	*Professionals* (General practitioners)	168	F = 33; M = 135	NA ^§^	NA	9
Semeah et al., 2017	US	Lifetime prevalence	*Professionals* (Nurses, other allied health professional)	35	NA	NA ^§^	NA ^§^	8
Wong et al., 2017	Canada	Lifetime prevalence	*Professionals* (Nurses)	823	F = 782; M = 41	48.6 (10.9)	12.2 (7.4)	8
Green and Ayalon, 2018	Israel	Lifetime prevalence	*Paraprofessionals* (Home healthcare aides)	523	F = 454; M = 69	43.9 (9.3)	NA	8
Ridenour et al., 2019	US	12-month prevalence	*Paraprofessionals* (Home healthcare aides)	513	F = 420; M = 25	NA ^§^	NA	9
Fujimoto et al., 2019	Japan	12-month + lifetime prevalence	*Professionals* (Nurses)	184	F = 176; M = 8	45.9 (9.3)	19.4 (8.0)	8

* Presence of missing data; ^§^ presence of categorized data. F = Female; M = Male; SD = Standard deviation; NA = Not available.

## Supplementary Materials

The following are available online at https://www.mdpi.com/1660-4601/17/23/8807/s1, Table S1: PubMed search strategy; Table S2: Included article quality assessment; Figure S1: Funnel plot and the Doi plot.
