# Supplementary material for: Prevalence of Workplace Sexual Violence against Healthcare Workers Providing Home Care: A Systematic Review and Meta-Analysis
Source: Int J Environ Res Public Health. 2020 Nov 27;17(23):8807. doi: 10.3390/ijerph17238807 (PMC7731391; doi:10.3390/ijerph17238807)
Supplement: Supplementary file 1 [file ijerph-17-08807-s001.zip › IHERPH Figure S1.docx]

**Figure S1a.** Funnel plot of the prevalence meta-analysis of workplace sexual violence against healthcare workers providing home care detecting major asymmetry.

**Figure S1(a)**

**Figure S1b.** Doi plot of the prevalence meta-analysis of workplace sexual violence against healthcare workers providing home care detecting major asymmetry.

**Figure S1(b)**
