# Supplementary material for: Prevalence of Workplace Sexual Violence against Healthcare Workers Providing Home Care: A Systematic Review and Meta-Analysis
Source: Int J Environ Res Public Health. 2020 Nov 27;17(23):8807. doi: 10.3390/ijerph17238807 (PMC7731391; doi:10.3390/ijerph17238807)
Supplement: Supplementary file 1 [file ijerph-17-08807-s001.zip › IJERPH Table S1.docx]

| **Table S1**. PubMed search strategy | | |
| --- | --- | --- |
| **Database** | **Search string** | **Number of records** |
| PubMed | (“Aggression”[Mesh] OR “aggression*” OR “Violence”[Mesh] OR “violence*” OR “Workplace Violence”[Mesh] OR “Workplace Violence*” OR “Physical Abuse*” OR “abuse*” OR “Sex Offense*” OR “Occupational Injuries”[Mesh] OR “Occupational Injury” OR “Occupational Injuries”) AND (“Physicians”[Mesh] OR “Physician*” OR “Medical Staff” OR “Health Personnel”[Mesh] OR “Health Personnel” OR “Healthcare worker*” OR “Health-care worker*” OR “Health employee*” OR “Healthcare employee*” OR “nurses”[Mesh] OR “nurse*” OR “caregivers”[Mesh] OR “caregiver*” OR “care-giver*” OR “case managers”[Mesh] OR “case manager*” OR “GP” OR “general practitioner*” OR “auxiliary assistant” OR “auxiliary nurse*” OR “nurse assistant*” OR “home care assistant*” OR “home-care assistant*” OR “home carer*” OR “social care worker*” OR “social care assistant” OR “social worker*” OR “community worker*” OR “nursing aid*” OR “Pharmacist*”) AND (“care home*” OR “nursing home*” OR “palliative care”[Mesh] OR “palliative care” OR “home care*” OR “home-care*” OR “hospital at home” OR “hospices”[Mesh] OR “hospice*” OR “retirement home*” OR “rest home*” OR “assisted living” OR “assisted-living” OR “home therapy” OR “home drug therapy” OR “home community care*” OR “pharmac*” OR “home care services”[Mesh] OR “home care service*” OR “home nursing”[Mesh] OR “home nursing”) | 4138 |
| Limitations: Published from inception to 29-02-2020 | | |
|  | | |
