# Supplementary material for: Prevalence of Workplace Sexual Violence against Healthcare Workers Providing Home Care: A Systematic Review and Meta-Analysis
Source: Int J Environ Res Public Health. 2020 Nov 27;17(23):8807. doi: 10.3390/ijerph17238807 (PMC7731391; doi:10.3390/ijerph17238807)
Supplement: Supplementary file 1 [file ijerph-17-08807-s001.zip › IJERPH Table S2.docx]

| **Table S2.** Included article quality assessment | | | | | | | |
| --- | --- | --- | --- | --- | --- | --- | --- |
| **Study** | **Q1** | **Q2** | **Q3** | **Q4** | **Q5** | **Q6** | **Total (Max: 11)** |
| Barling et al (2001) | 1 | 1 | 2 | 3 | 2 | 2 | 11 |
| Astrom et al (2002) | 1 | 0 | 2 | 3 | 2 | 1 | 9 |
| Ayalon (2009) | 1 | 0 | 2 | 3 | 2 | 0 | 8 |
| Nakaishi et al (2013) | 1 | 0 | 2 | 3 | 2 | 0 | 8 |
| Hanson et al (2015) | 1 | 0 | 2 | 3 | 2 | 1 | 9 |
| Green & Ayalon (2016) | 1 | 0 | 2 | 3 | 2 | 0 | 8 |
| Quinn et al (2016) | 1 | 0 | 2 | 3 | 2 | 1 | 9 |
| Fujimoto et al (2017) | 1 | 1 | 2 | 3 | 1 | 1 | 9 |
| Ifediora (2017) | 1 | 0 | 2 | 3 | 2 | 1 | 9 |
| Semeah et al (2017) | 1 | 0 | 2 | 3 | 2 | 0 | 8 |
| Wong et al (2017) | 1 | 0 | 2 | 3 | 2 | 0 | 8 |
| Green & Ayalon (2018) | 1 | 0 | 2 | 3 | 2 | 0 | 8 |
| Ridenour et al (2019) | 1 | 0 | 2 | 3 | 2 | 1 | 9 |
| Fujimoto et al (2019) | 1 | 0 | 2 | 3 | 1 | 1 | 8 |
